# Surgical Removal of a Long-Standing Impacted Firearm in Neck: A Case Report

**DOI:** 10.31729/jnma.7327

**Published:** 2022-04-30

**Authors:** Arun Adhikari, Nain Bahadur Mahato, Bijay Khatri

**Affiliations:** 1 Kantipur Hospital, Tinkune, Kathmandu, Nepal; 2 B.P. Eye Foundation, Hospital for Children, Eye, ENT and Rehabilitation Services, Lokanthali, Bhaktapur, Nepal

**Keywords:** *case reports*, *firearms*, *foreign body*, *neck*

## Abstract

The gunshot neck injury is an emergency and must be addressed immediately. However, as our case report, a long-standing retained foreign body (firearm) in the deep neck is rare. Surgical removal of a long-standing foreign body is challenging for the operating surgeon. A 36-year-old male with a history of a firearm injury to the neck dating 16 years back with complaints of recent onset of pain was evaluated. A computed tomography scan of the neck showed a metallic foreign body located in close proximity to the right common carotid artery. Neck exploration was performed under general anaesthesia, and the foreign body was removed without complications. The patient has recovered following the intervention and has resumed his normal activities. The use of methylene blue helps to locate the foreign body during surgery. However, the choice of intervention for a stable patient with a penetrating neck injury remains based on cases.

## INTRODUCTION

A decade-long armed conflict in Nepal killed thousands of people, and many more suffered from ballistic injuries. Most ballistic injuries were managed immediately by removing the foreign body, and unmanaged ones result in complications or dormant embedding in soft tissues. This case report describes the surgical management of a ballistic foreign body stuck in deep neck space for 16 years. With firearm issues being a global public health problem^1^ and never-ending armed conflicts worldwide,^2^ such cases may arise in the future.

## CASE REPORT

A former insurgent, 36-year-male, presented himself at the Ear Nose Throat (ENT) Out Patient Department (OPD) of a tertiary care centre in Kathmandu, with complaints of pain in deep neck space for the last four months, which was radiating to jaws, for which he was taking painkillers for the last five days. He reported that he had sustained a firearm injury during the later years of armed conflicts in Nepal. Immediately after the injury, the health workers accompanying him tried to remove the foreign body, but the removal was not performed due to the unavailability of proper instruments. Hence, he was managed with some painkillers and antibiotics. The following day, swelling around the wound developed, and the removal was not attempted. Later the wound healed, and as there was no pain or any other difficulties for the patient, to seek expert advice for more than 16 years.

On initial inspection and physical examination at OPD, there was a stony hard foreign body at the level of the right thyroid lamina, which adhered to the underlying structure and was free from the overlying skin. The scar mark of the entry point was present just above and medial to the foreign body. Some firearm particles were also present on the anterior aspect of his left leg, volar aspect of the right hand, and centre of the forehead, where scar marks were visible, but as he has no symptoms, he does not want to address them now. He didn't report any drug allergies. Stroboscopy was performed to rule out any abnormality in the vocal cords, which showed no abnormality. A computed tomography scan of the neck showed a hyperdense linear structure measuring 15x6.4 mm in the right cervical region, just lateral to the laminae of thyroid cartilage on the right side. It lied anterior to the right common carotid artery. A hyperdense halo surrounded it with a maximum width of 3 mm, adjacent to the right laminae of thyroid cartilage showing scalloped margin and sclerotic changes.

The patient and the patient party were counselled that the foreign body might not be removed if it could not be localized or if found to have adhered to the vital structures as injury to vital structures may lead to complications. After clearance from the local police authority and written consent from the patient and patient party, neck exploration and foreign body removal under general anaesthesia were planned. The pre-operative investigations of blood, electrocardiogram, and chest X-ray showed he was fit for surgery under general anaesthesia.

**Figure 1:**
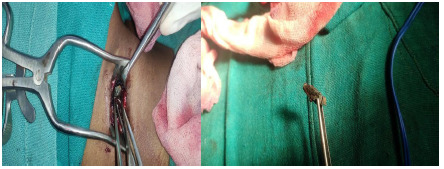
A) Firearm being removed intraoperatively, B) Firearm after removal.

Under general anaesthesia, the patient was placed in the supine position. Methylene blue dye was injected, directing towards the foreign body to demarcate the line of dissection. A horizontal skin crease incision was given and dissected till the surrounding fibrosed tissue. The fibrosed tissued was dissected, and the metallic foreign body was removed. Attempted removal of fibrosed tissue was not successful as the fibrosed tissue sac adhered to the right common carotid artery. Hence, only part of the sac was removed, leaving the portion adhered to the common carotid artery. The Penrose drain was placed, and the wound was closed in layers. The intervention lasted for around 1 hour and 45 minutes (Figure 1).

**Figure 2:**
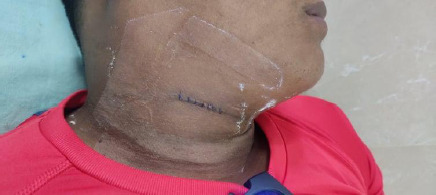
Lateral aspect of the neck on the first postoperative day after removal of the drain.

There were no complications or unanticipated events during the surgery. The drain was removed on the first postoperative day, and the patient was discharged on the second postoperative day (Figure 2).

The patient was advised to take antibiotics, painkillers, and anti-inflammatory drugs for a week, and sutures were removed on the seventh postoperative day. He was advised to take painkillers if required and asked to visit after a week following the suture removal. A fortnight after the surgery, the patient reported no pain and did not have any painkillers. No more medications were advised after that.

Four weeks following surgery, the patient reported resuming his daily chores during the follow-up visit. On follow-up, 14 weeks after surgery, the patient was doing well and had resumed his daily chores.

## DISCUSSION

The firearm neck injury is an ENT emergency and must be addressed immediately. Firearm injuries were common during the armed conflicts in Nepal, and most former insurgents are still living with firearms foreign bodies within them. Our case report is one of them who contacted us after 16 years following discomfort and pain near the injury area, neck. Patients who sustain firearm injuries to the head and neck region face heavy tissue damage and eventually life-threatening conditions. Knowledge of the path of the bullet and how it terminates is critical for expeditious assessment and optimal management of patients with firearm wounds. The extent of tissue damage depends on internal lacerations, tissue compression, and the temporary cavitation along the projectile path.^3^ In contrast to wounds caused by blow or impact to the viscerocranium, bullet wounds are characterised by an irregular path, entry and exit wounds, and localised demolition of bones with the associated effects.^3^

In our case, the injury dated back to December, 2004, the entry point scar was visible just above the point where the foreign body was palpable, but as the foreign body was impacted within the soft tissue, no exit wound was visible. The magnitude of the damage caused by any ballistic projectile is dependent on the target material and projectile parameters.^4^ In general, high-velocity missiles traverse the body linearly, penetrating the soft tissues and breaking the bones in the way.^5,6^ They also cause damage by creating cavities and producing stress waves.^7^ The skull base is especially vulnerable to the shock waves created by missile injuries to the head and neck.^8^ By contrast, low-velocity missiles damage the tissue through laceration and crushing, and they tend to remain within the superficial tissue planes.^9^ The patient claimed a direct hit by a bullet in our case. However, as the vital structures were not involved and he was able to contain the firearm for more than 16 years, it is possible that the firearm struck somewhere else outside the body and then injured his neck with decreased velocity.

The lateral neck is divided into three zones which is useful in evaluating and treating penetrating neck injuries.^10,11^ According to this classification, our case falls into zone 2 as the firearm was present just in front of the common carotid. Surgeons must be careful not to injure the common carotid or the recurrent laryngeal nerve while performing surgery.

We did not find a similar case reported in our literature search. In this case, we injected methylene blue directed toward the foreign body to delineate the line of dissection. This made our dissection easy, and we could reach the foreign body early without injuring the other surrounding structures. We also left the sac containing the foreign body behind to some extent to avoid injury to the common carotid artery as it adhered to it, and we believed that injury to the common carotid might lead to dreadful complications. This case illustrates that different factors are involved in the outcome of a firearm injury, among which ballistic factors and the site of the impacted foreign body are of utmost importance, and each of these types of injuries can be managed on a case basis.

The patient is satisfied post-operatively as he no longer needs to depend on painkillers and is without any symptoms. However, he is concerned that other firearm particles present on other parts of his body might exude pain in the future, and he may need to undergo surgery for them too. He says there are many other cases of impacted firearm injury among former insurgents in Nepal which are yet to be managed due to the unavailability of services within their reach or cannot afford the intervention. He bemoans the government's lack of support for his colleagues suffering from such injuries.

The impacted foreign body from a firearm can vary in its presentation at different parts of the neck or human body. The treatment of choice will depend on the clinical presentation of each case, and the use of methylene blue helped delineate the line of dissection. We also left the sac containing the foreign body behind to some extent to avoid injury to the common carotid artery as it adhered to it. Asymptomatic cases may carry the foreign body for a long duration without complications if vital structures are not damaged. There can be many other cases in Nepal that need timely intervention before they develop any complications and become unremovable.
